# Effects of High-Grain Diet on Performance, Ruminal Fermentation, and Rumen Microbial Flora of Lactating Holstein Dairy Cows

**DOI:** 10.3390/ani14172522

**Published:** 2024-08-30

**Authors:** Kexin Wang, Damin Song, Xuelei Zhang, Osmond Datsomor, Maocheng Jiang, Guoqi Zhao

**Affiliations:** 1 Institute of Animal Culture Collection and Application, College of Animal Science and Technology, Yangzhou University, Yangzhou 225009, China; awkx5527@163.com (K.W.); songdm0713@163.com (D.S.); 15056282672@163.com (X.Z.); datsomorosmond@gmail.com (O.D.); 2 Institutes of Agricultural Science and Technology Development, Yangzhou University, Yangzhou 225009, China; 3 Joint International Research Laboratory of Agriculture and Agri-Product Safety, The Ministry of Education of China, Yangzhou University, Yangzhou 225009, China

**Keywords:** high-grain diet, rumen fermentation, bacterial community composition

## Abstract

**Simple Summary:**

This study indicates that a high grain diet (HG) significantly reduces the milk fat content of lactating cows. In addition, the HG group promoted rumen fermentation by altering the microbial composition of lactating cows. The concentration of short-chain fatty acids (SCFAs) in the HG group was increased by increasing the abundance of *Prevotella* and *Xanthomonas*, providing more energy to the body, while increasing the abundance of *Stenotrophomonas*, which increased the probability of mastitis. However, 16S sequencing still cannot reflect the true physiological changes in the host. Therefore, future research should investigate the effects between diet, microorganisms, and hosts to facilitate managers to use high-grain diets rationally to achieve high yields.

**Abstract:**

The objectives of the current study were to evaluate the fluctuations in production performance, rumen fermentation, and microbial community in lactating dairy cows fed a high-grain diet (HG). In this study, 16 healthy Holstein lactating dairy cattle with similar milk yields of 16.80 ± 4.30 kg/d, days in milk 171.44 ± 23.25 days, and parity 2.2 ± 1.5 times were selected and randomly allocated into two groups. One group was fed a low-grain diet (LG; 40% concentrate, DM basis; n = 8), and the other group was fed a high-grain diet (HG; 60% concentrate, DM basis; n = 8). The experiment lasted 6 weeks, including 1 week for adaptation. The experimental results showed that the milk fat content in the milk of lactating cows in the HG group was significantly reduced (*p* < 0.05), and the milk urea nitrogen (MUN) content showed an increasing trend (0.05 < *p* < 0.10) compared with the LG group. Compared with the LG group, rumen fluid pH was significantly decreased after feeding a high-grain diet, and contents of total volatile fatty acids (TVFA), acetate, propionate, and butyrate were significantly increased (*p* < 0.05). The acetate/propionate significantly decreased (*p* < 0.05). HG group significantly increased the abundance of *Prevotella* and *Bacteroides* in rumen fluid while significantly reducing the abundance of *Methanobrevibacter* and *Lachnospiraceae ND3007_group* (*p* < 0.05). Microorganisms with LDA scores > 2 were defined as unique, with the bacterial genus *Anaerorhabdus_furcosa_group* identified as a biomarker for the LG group, and the unique bacterial genus in the HG group were *Prevotella*, *Stenotrophomonas*, and *Xanthomonadaceae*. The prediction results of microbial function showed that a total of 18 KEGG differential pathways were generated between the two treatment groups, mainly manifested in metabolic pathways, signal transduction, and the immune system. In conclusion, the HG group promoted rumen fermentation by altering the microbial composition of lactating cows. Our findings provide a theoretical basis for the rational use of high-grain diets to achieve high yields in intensive dairy farming.

## 1. Introduction

High-grain diets (HG) are often fed to high-producing lactating cows to increase milk production. Overfeeding HG often leads to subacute rumen acidosis (SARA) in lactating cows. In past studies, it has been found that the production performance and rumen fermentation of lactating cows can be affected by the occurrence of SARA [1]. At the same time, SARA greatly endangers the health of cows, increases the elimination rate of cows, and thus has a negative impact on the profitability of farms [2]. However, continuous feeding of HG will lead to abnormal rumen fermentation in dairy cows and copious short-chain fatty acids (SCFAs) produced in the rumen. When organic acids exceed the absorption and buffering capacity of the rumen, they will accumulate in the rumen, resulting in decreased pH in the rumen and disturbance of the flora, ultimately affecting the health of dairy cows [3]. In other ruminants, evidence suggests that long-term feeding of high-grain diets can change rumen fermentation patterns and microbial communities [4].

The rumen microbiota plays a crucial role in animal nutrient absorption. The changes in rumen microbiota positively correlate with rumen fermentation efficiency and feed utilization efficiency in ruminant production [5]. Furthermore, the structure and abundance of the microbial community in the rumen are also influenced by various factors, such as antibiotics and feed constitution [6]. A study has found that the diversity and composition of fecal microbiota in cows alter during SARA conditions, which are directly related to the health of cows [7]. In addition, studies have found that changes in the rumen microbiota can affect the host’s metabolism, immune response, and other physiological responses, such as lipopolysaccharide (LPS) and SCFAs, through bacterial decomposition or metabolic products [8].

Despite extensive research on SARA, it is still unclear how feeding HG affects the rumen microbial homeostasis of lactating cows. The study hypothesized that feeding a high-grain diet can affect the performance, ruminal fermentation, and rumen microbial flora of lactating Holstein dairy cows. Therefore, the objectives of the current study were to evaluate the fluctuations in production performance, ruminal fermentation, and microbial community in lactating dairy cows fed a high-grain diet.

## 2. Materials and Methods

This experiment was conducted on the experimental farm (Jiangsu, China) in March 2023, and the animal feeding and care guidelines (SYXK (Su) 2021-0026) followed the Institutional Animal Care and Use Committee (IACUC) of Yangzhou University.

### 2.1. Experimental Design and Animal Management

Sixteen healthy Holstein lactating dairy cows with similar milk yields of 16.80 ± 4.30 kg/d, days in milk 171.44 ± 23.25 days, and parity 2.2 ± 1.5 times were selected and randomly allocated into two groups. One group was fed a low-grain diet (LG; 40% concentrate, DM basis; n = 8) and the other group was fed a high-grain diet (HG; 60% concentrate, DM basis; n = 8). The experiment lasted 6 weeks, including 1 week for adaptation. The diet formula used in the experiment was configured according to the nutritional requirements of dairy cows by the National Research Council (NRC) [9], and the LG and HG groups were set to equal energy and nitrogen diets. All the dairy cows were fed the total mixed ration diet (TMR), which was formulated according to Table 1. Cows were raised in a tethered manner and had free access to water and feed. They were fed approximately 105% of the daily intake in three separate portions (07:00, 13:00, and 20:00 h).

### 2.2. Sample Collection and Analyses 

The feeding amount and remaining amount are recorded on the last three consecutive days of each week to evaluate lactating cows’ dry matter intake (DMI). Feed samples were collected and placed in an oven to remove moisture (65 °C, 48 h) and then the dried samples were ground using a grinder (2 mm mesh, model 1188Y, Thomas Willey, NJ, USA). The sample was tested for nutritional components using the method proposed by Jiang et al. [10]. CP was assessed using the Scino KT260 method (FOSS, Hillerod, Denmark); NDF and ADF were analyzed using a fiber optic analyzer (model 2000i, Ankom, Macedon, NY, USA) based on Van Soest’s method; Starch was determined by the Solarbio starch content detection kit (#BC0705, Solarbio, Beijing, China) and ash content by the AOAC method 942.05. 

Milk samples were collected per Jiang’s method [8] on the last 3 days of each week and samples were mixed from three-time points daily in a 4:3:3 ratio and preserved before submission to Dairy One Cooperative Inc., Shanghai, China, for composition analysis. The energy-corrected milk (ECM) value was computed using Jiang’s formula [11], as follows:

(1) $$ECM\left(kg/cow\;per\;day\right)=milk\;yield(kg/day)\times \frac{376\times fat\%+209\times protein\%+948}{3138}$$

According to Wolff’s method [12], after eating for 3 h in the morning, collect rumen fluid 50 mL using an oral gastric tube from 6 cows in each group. The rumen fluid sample should be filtered through four layers of gauze to detect pH value (PB-21, Beijing Sartorius Scientific Instrument Co., Ltd., Beijing, China) and then stored in a −20 °C refrigerator. The content determination of volatile fatty acid (VFA) followed the previous experimental method [13] is quantifiable using gas chromatography (Thermo Fisher Scientific, Waltham, MA, USA).

### 2.3. DNA Extraction and 16S rRNA Gene Sequencing

DNA was extracted from 1 mL rumen fluid with the DNA extraction kit (Tiangen Biotech, Beijing, China) and purified DNA was eluted in 200 µL of eluent. The NanoDrop ND-2000 spectrophotometer (Thermo Fisher, Waltham, MA, USA) was used to determine the concentration of DNA, and 2% agarose gel electrophoresis was used to evaluate the DNA quality. Specific primers were used to amplify the 16s rDNA and V3-V4 region from the obtained DNA. The primer sequence [4] is 341 F: CTACGGGNGGCWGCAG and 806 R: GGACTACHVGGGTATCTAAT. The PCR reaction was performed twice with a volume of 50 µL. All PCR reactions were performed in a 50 µL reaction containing 50 ng of extracted template DNA, 1.5 µL of each forward and reverse primers (10 µM), 1.5 µL of dNTP (2.5 mM each), 10 µL of 5× Q5@ Reaction Buffer (Vazyme Biotech Co., Ltd., Nanjing, China), 10 µL of 5× Q5@ High GC Enhancer (Vazyme Biotech Co., Ltd., Nanjing, China), and 0.2 µL of Q5@ High-Fidelity DNA Polymerase (Vazyme Biotech Co., Ltd., Nanjing, China). The thermal cycling procedures were as follows: initial denaturation at 95 °C for 5 min; 30 cycles of denaturation (95 °C, 1 min), annealing (60 °C, 1 min), and elongation (72 °C, 1 min); and a final extension at 72 °C for 7 min. Finally, the construction and implementation of the sequencing library followed the method of Zhang et al. [4] In short, the quality of the isolated DNA was assessed on a 1% agarose gel, and 300 bp DNA was fragmented using Covaris M220 (Gene Company Limited, Hong Kong, China) to construct paired-end libraries. Paired-end libraries were generated using NEXTFLEX Rapid DNA-Seq (Bio Scientific, Austin, TX, USA). The ligate adapters carry the full sequencing primer hybridization site to the blunt ends of the fragments. Paired-end sequencing was performed on Illumina NovaSeq/Hiseq Xten (Illumina Inc., San Diego, CA, USA) from Genedenovo Biotechnology Co., Ltd. (Guangzhou, China) with NovaSeq Reagent Kits/HiSeq X Reagent Kits following operational guidelines (www.illumina.com, accessed on 27 July 2023).

### 2.4. Bioinformatics Analysis of the Sequence Data

Using the previous data processing method [14], the raw data were filtered employing the FASTP (version 0.18.0) to obtain high-quality clean reads. The clean reads thus obtained were clustered into operational taxonomic units (OTUs) of ≥97% similarity using UPARSE (version 9.2.64) [15]. Meanwhile, effective reads are filtered through the UCHIME algorithm [16].

The classification was performed using BLAST (version 2.6.0) in the NCBI 16S ribosomal RNA database (Bacteria and archaea) (http://www.ncbi.nlm.nih.gov, accessed on 25 July 2023). The search for representative OTU in version 202101 followed the classification method of Altschul et al. and filtered different categories [17]. The reserved sequences without BLAST hits were marked as unclassified. The visualization of the abundance statistics of various taxa was conducted using Krona (version 2.6) [18]. The stacked bar chart of microbial composition was visualized and analyzed using the R project ggplot2 package (version 2.2.1) [19]. The linear discriminant analysis effect size (LEfSe) of the rumen bacterial community was used to analyze specific bacterial communities in the experimental group [20]. Classification of bacterial microbiome phenotypes was performed via BugBase [21]. To elucidate the impact of fermentation parameters on community composition, the R project Vegan package (version 2.5.3) [22] was utilized. The Spearman correlation coefficient between fermentation parameters and species was calculated using the R project psychology package (version 1.8.4) [23]. For a deeper understanding of microbial functionality, we employed PICRUSt2 v2.4.1 (https://github.com/picrust/picrust2/wiki, accessed on 27 October 2023) to predict functional profiles based on 16S rRNA gene sequences, which were represented as Kyoto Encyclopedia of Genes and Genomes (KEGG) [24]. The generation of heat maps and correlation coefficient networks was conducted using the dynamic real-time interactive online data analysis platform (Omicsmart, http://www.omicsmart.com, accessed on 27 October 2023). The data obtained in this study were submitted to the National Center for Biotechnology Information (NCBI) under submission ID: SUB 14629327 and BioProject ID: PRJNA1141126.

### 2.5. Statistical Analysis 

The data are presented as means ± standard error of the mean (SEM). Statistical analyses were performed using the Statistical Package for the Social Sciences (SPSS) version 20.0 (IBM Corp., Armonk, NY, USA), utilizing ANOVA and the unpaired Student’s *t*-test [25]. *p*-values less than 0.05 were deemed statistically significant, while values between 0.05 and 0.10 indicated a trend.

## 3. Results

### 3.1. Feed Intake and Lactation Performance

Compared with the LG group, the HG group significantly decreased milk fat content in the milk of lactating cows (Table 2, *p* < 0.05) but had no significant effects on DMI and milk yield of lactating cows (*p* > 0.05) and had a tendency to increase the MUN content (0.05 < *p* < 0.10).

### 3.2. Ruminal Fermentation Characteristics

Compared with the LG group, rumen fluid pH in lactating cows was significantly decreased after feeding a high-grain diet, and the contents of TVFA, acetate, propionates, and butyrate were significantly increased (Table 3, *p* < 0.05). It is worth noting that compared with the LG group, the mole percentage of acetate significantly decreased, while the mole percentage of propionate significantly increased in the HG group (*p* < 0.05). The acetate/propionate significantly decreased (*p* < 0.05), but the contents of NH_3_-N, isobutyrate, valerate, and isovalerate had no significant effect (*p* > 0.05).

### 3.3. Sequencing and Diversity Measures

**Figure 1:**
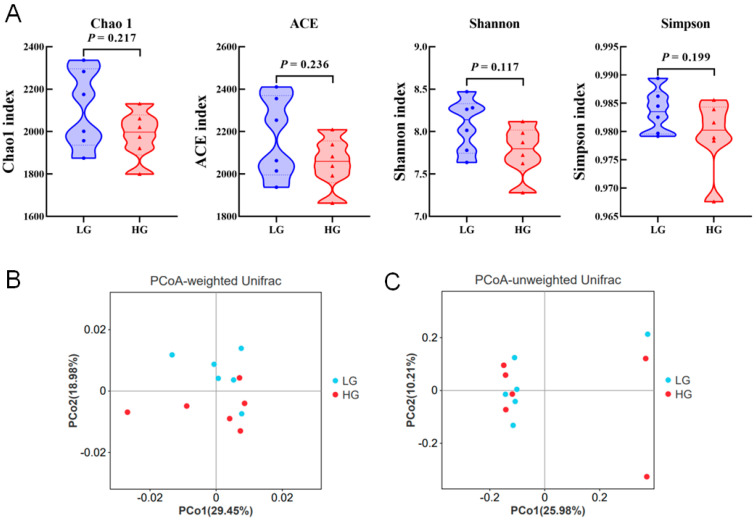
Principal coordinate analysis (PCoA) and α-diversity indexes of bacterial community structure between the LG and HG treatments. (**A**) α-diversity indexes; (**B**) PCoA based on weighted Unifrac matrix; (**C**) PCoA based on unweighted Unifrac matrix. LG = Low-grain diet; HG = High-grain diet.

High-quality V3-V4 16S rRNA sequences of 12 samples generated a total of 1,244,027 raw reads pairs, and 1,243,101 reads pairs passed through quality filtering and host-genome removal with an average number of 103,592 reads per sample. The high-quality reads pairs ratio among all the raw reads pairs from each sample was 78.01% on average (Supplementary Table S1). The Venn diagram shows the number of common and unique rumen bacterial OTUs in two groups (Supplementary Figure S1). The ROC curve analysis of OTUs in rumen samples showed that the sequencing results were sufficient to accurately describe the bacterial communities of the two groups (Supplementary Figure S2). The α diversity was compared between two groups of cows using the ACE index, Chao1 index, Shannon index, and Simpson index. The results showed no significant effect between the LG and HG treatment groups (Figure 1A, *p* > 0.05). For the β diversity of rumen fluid bacterial communities between the two treatments, we conducted PCoA analysis based on weighted and unweighted Unifrac distances (Figure 1B,C). 

### 3.4. Ruminal Microbiota Composition

**Figure 2:**
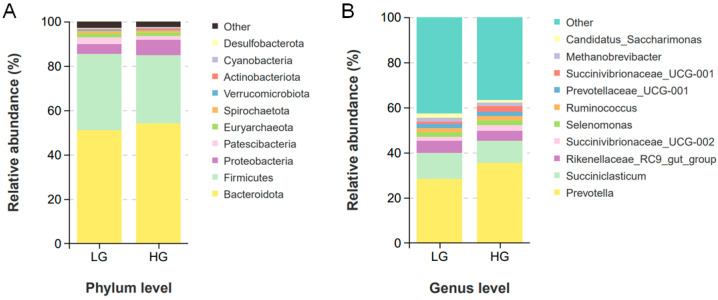
Distribution of rumen bacterial community composition in LG and HG groups. (**A**) Phylum level; (**B**) genus level. LG = Low-grain diet; HG = High-grain diet.

**Figure 3:**
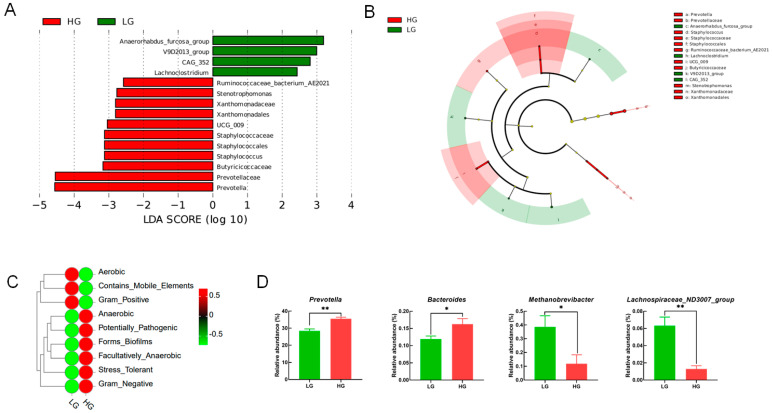
The linear discriminant analysis effect size (LEfSe) of the changes in the rumen bacterial community between LG and HG groups. (**A**) LDA score. The LDA score was derived from the LEfSe analysis, which showed the biomarker taxa LDA score > 2 of rumen microbiota in LG and HG groups; (**B**) LG and HG group cladogram. A cladogram showing the relationships among taxa at phylum, class, order, family, and genus levels was generated using LEfSe analysis (n = 6 per group); (**C**) Predicting bacterial phenotype abundance heatmap. (**D**) The significant differential rumen bacteria affected between LG and HG groups. LG = Low-grain diet; HG = High-grain diet. * Indicates 0.01 < *p* ≤ 0.05, ** indicates *p* ≤ 0.01.

The effect of different dietary compositions on rumen microbial composition was analyzed by classifying the microbial community in rumen fluid. A total of 26 bacterial phyla were obtained from 12 rumen fluid samples, including *Bacteroidota* (LG: 51.00 ± 1.55%; HG: 54.11 ± 1.95%), *Firmicutes* (LG: 34.37 ± 1.60%; HG: 30.70 ± 1.34%), *Proteobacteria* (LG: 4.42 ± 0.76%; HG: 6.86 ± 2.25%), and *Patescibacteria* (LG: 3.02 ± 0.92%; HG: 1.71 ± 0.20%), were the major clades in all groups (Figure 2A). At the genus level, a total of 276 genera were detected in the rumen. The main dominant genera were *Prevotella* (Figure 2B, LG: 28.42 ± 1.02%; HG: 35.50 ± 0.84%), *Succiniclasticum* (LG: 11.45 ± 0.47%; HG: 9.72 ± 0.95%), and *Rikenellaceae_RC9_gut_group* (LG: 5.33 ± 0.12%; HG: 4.45 ± 0.65%). This experiment found that a high-grain diet significantly increased the abundance of *Prevotella* and *Bacteroides* in rumen fluid while significantly reducing the abundance of *Methanobrevibacter* and *Lachnospiraceae ND3007_group* (Figure 3D, *p* < 0.05). In order to identify biomarkers of specific microorganisms in rumen fluid after two dietary treatments, the LEfSe selection was used to analyze the data (Figure 3A), and a branching plot was generated through LEfSe analysis of rumen microbial communities (Figure 3B). Microorganisms with LDA scores > 2 were defined as unique, with the bacterial genus *Anaerorhabdus_furcosa_group* identified as a biomarker for the LG group, and the unique bacterial genus in the HG group were *Prevotella*, *Stenotrophomonas*, and *Xanthomonadaceae*. At the same time, we predicted the bacterial phenotype of rumen microbial communities after two dietary treatments. The results showed that compared with the LG group, the abundance of anaerobic and Gram-negative bacteria in the HG group was significantly increased, while the abundance of Gram-positive bacteria was decreased (Figure 3C). Compared with the LG group, the HG group significantly increased the abundance of *Prevotella* and *Bacteroides* in rumen fluid, while reducing the abundance of M and L

### 3.5. Correlation Analysis of Rumen Fermentation Parameters and Major Bacteria

**Figure 4:**
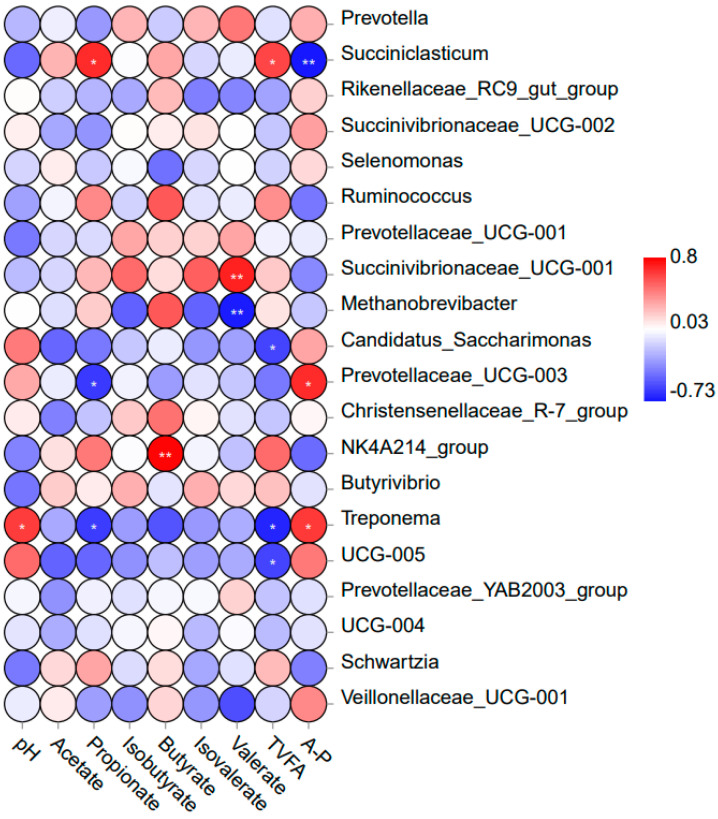
Spearman correlation between rumen fermentation characteristics and rumen microbiota (genus level). Red indicates a significant positive correlation (*p* < 0.05), while blue indicates a significant negative correlation (*p* < 0.05). A-P = Acetate/propionate. * Indicates 0.01 < *p* ≤ 0.05, ** indicates *p* ≤ 0.01.

Spearman correlation analysis was conducted to evaluate the relationship between rumen fermentation parameters and the structure of the rumen microbial community across different dietary compositions (Figure 4). At the genus level, the relative abundance of *Succiniclasticum* was negatively correlated with A/P (r = −0.73; *p* < 0.01) and positively correlated with TVFA (r = 0.61; *p* < 0.05) and propionate (r = 0.69; *p* < 0.05). The relative abundance of *Succinivibrionaceae_UCG-001* was positively correlated with valerate (r = 0.71; *p* < 0.01). The relative abundance of *Methanobrevibacter* was negatively correlated with valerate (r = −0.72; *p* < 0.01). The relative abundance of *Candidatus_Saccharimonas* was negatively correlated with TVFA (r = −0.59; *p* < 0.05). The relative abundance of *Prevotellaceae_UCG-003* was negatively correlated with propionate (r = −0.63; *p* < 0.05) and positively correlated with A/P (r = 0.69; *p* < 0.05). The relative abundance of the *NK4A214_group* was positively correlated with butyrate (r = 0.80; *p* < 0.01). The relative abundance of *Treponema* was negatively correlated with TVFA (r = −0.69; *p* < 0.05) and propionate (r = −0.62; *p* < 0.05) and positively correlated with pH (r = 0.64; *p* < 0.05) and A/P (r = 0.64; *p* < 0.05). The relative abundance of *UCG-005* was negatively correlated with TVFA (r = −0.59; *p* < 0.05).

### 3.6. Functional Prediction of the Microbial Community Structure

**Figure 5:**
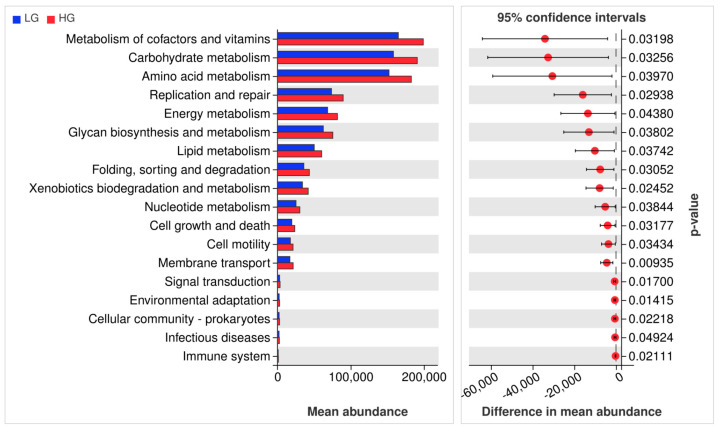
Predicted functional profile of PICRUSt2 in microorganisms. LG = Low-grain diet; HG = High-grain diet.

Predictions of functional abundance based on OTUs were generated using PICRUSt2 to assess the functional potential of microorganisms. It is worth noting that our research results showed a total of 18 KEGG differential pathways, all of which were significantly enriched in the HG group (Figure 5). The results indicate that 8 KEGG differential pathways belong to metabolic processes, mainly including the metabolism of cofactors and vitamins, carbohydrate metabolism, amino acid metabolism, energy metabolism, Glycon biosynthesis and metabolism, and Lipid metabolism. Some functions are mainly focused on cellular processes and signal transduction. It is worth noting that there are significant differences in the enrichment of immune system functionality between the two treatment groups.

## 4. Discussion

In the process of managing cows, using HG to improve their lactation performance is one of the common approaches. Long-term high-grain diets can cause a series of nutritional metabolic diseases and economic losses to lactating cows [26]. High grains that are easy to ferment will quickly degrade into SCFAs in the rumen, reducing the pH value of rumen fluid, which is not conducive to the growth and reproduction of microorganisms and can cause microbial community disorder [27]. Therefore, this experiment aims to explore the effects of high-grain diets on the lactation performance, rumen fermentation parameters, and microbial community of lactating cows.

In this experiment, the structure of the diet was changed by adjusting the ratio of grain in the diet, and the characteristics of high carbohydrate corn were used as the main source of a high grain diet. The starch content of LG and HG were different due to the different proportions of corn added (17.36% vs. 24.72%). In previous research reports, high starch diets fermented in the rumen produce more propionic acid, which increases lactose content through glucose metabolism [28]. On the other hand, the difference in lactose production may, to some extent, lead to differences in milk production among cows fed with high and low starch-content diets, as lactose is the main osmotic regulator that controls milk production [29,30]. However, in this experiment, there was no change in milk production and lactose content due to dietary treatment. Cui et al.’s study [31] used diets with a forage-to-concentrate ratio of 4:6 and 6:4. Compared with the control group, the high-concentrate feed group did not significantly improve the DMI and milk production of lactating cows, which is similar to the results of this experiment. The intake of dairy cows is regulated by two main mechanisms: chemical static regulation and physical regulation (rumen distension caused by rumen filling) [30]. In this experiment, the regulatory effect of rumen distension decreased with the reduction in coarse feed in TMR, which may change the filling degree of rumen and reduce rumen distension. At this time, physical regulation shifted to chemical static regulation as the dominant factor. Therefore, feeding HG did not change the dietary intake of lactating cows. Milk fat content is an essential indicator for measuring the quality of lactating milk [32]. The results of this experiment showed that feeding a high-grain diet significantly reduced the fat content in cow milk. A high-grain diet can lead to higher concentrations of SCFAs in the rumen and a decrease in the ethylene-propylene ratio and promote the transformation of rumen fermentation into propionic acid fermentation [31]. Propionic acid is a source of gluconeogenesis, which generates a large amount of glucose in the body, leading to an increase in insulin secretion and the accumulation of a large amount of fatty acids in the blood into adipose tissue. As a result, the body fat content is increased, while the fatty acids used for synthesizing milk fat are reduced, decreasing milk fat percentage. Usually, the concentration of milk urea nitrogen is influenced by dietary crude protein intake and digestibility [33]. When cows experience SARA, sustained low pH levels can affect the utilization of ammonia by rumen microorganisms, leading to an increase in MUN [34]. This may be one of the reasons why the HG group MUN exceeds the normal range.

The main energy source for ruminants is SCFAs produced by rumen microorganisms. However, increased fermentable carbohydrates and decreased fiber intake in high-grain diets can cause subacute acidosis in the rumen, accumulating SCFAs. This can also affect the chewing ability, saliva buffer supply, and rumen motility of cows [2]. Rumen pH detection is a common indicator for diagnosing SARA [35]. After treatment with a high-grain diet in this experiment, the rumen pH was significantly lower than that of the LG group, possibly due to the diet’s lower NDF concentration and higher starch content. Compared with the LG group, the HG group often had higher concentrations of rumen TVFA, acetate, propionate, and butyrate in terms of the content of SCFAs in rumen fluid. The high starch content in high-grain diets promotes the production of more propionic acid in the rumen, leading to a significant decrease in the acetate/propionate ratio and a tendency toward propionic acid fermentation. The stability of the rumen environment is often influenced by the pH of the rumen fluid and fermentation parameters [36]. The changes in rumen pH value, to some extent, reflect the health status of the rumen [37]. The increase in SCFA concentration in rumen fluid often results in lower pH values, which can affect the normal function of rumen microorganisms [38]. The changes in dietary composition have significantly altered the pH and fermentation parameters of rumen fluid; however, the impact of long-term feeding of HG on rumen microbiota remains unknown. Therefore, the rumen microbial communities in the two diets were analyzed.

In this study, we collected rumen fluid samples from cows fed an HG for 40 days and revealed the bacterial composition in the rumen at the phylum and genus levels. Bacteroidetes and Firmicutes are the most dominant bacterial phyla at the phylum level, followed by *Proteobacteria*, *Patescibacteria, Euryarchaeota, Spirochaetota,* and *Verrucomicrobiota*. The Prevotella, Succiniclasticum, and Rikenellaceae_RC9_gut_group were identified as the main dominant genera at the genus level. This result indicates that *Firmicutes*, *Bacteroidetes,* and *Proteobacteria* are the main core microbial communities in the rumen fluid of cows, consistent with previous reports [39]. In addition, the abundance of *Firmicutes* and *Bacteroidetes* accounts for approximately 85% of the rumen fluid microbiota (at the phylum level), while the remaining bacteria only account for a small proportion. These results may also suggest that rumen contents can affect the abundance of rumen fluid bacteria, and this experiment provides a basis for further studying the changes in rumen bacterial abundance influenced by diet. Grains that are easy to ferment will be metabolized into SCFAs in the rumen, while the rumen with absorption function will absorb these substances and provide energy for the body. Previous studies have shown that the structure and composition of rumen bacteria are influenced by host, diet, and antibiotics [40]. Among these possible influencing factors, diet may be one of the main factors affecting bacterial structure and function [41]. The microbial community composition in this experiment showed significant differences in some bacterial communities between the HG and LG groups. The impact on microbial communities includes a decrease in their richness and diversity, which may reduce their function and reflect a corresponding imbalance in microbial homeostasis [42]. However, diet did not affect bacterial richness (ACE and Chao 1) and species diversity (Shannon and Simpson). Meanwhile, this also indicates that the HG group treatment has no significant effect on the microbial homeostasis of lactating cows. In addition, PCoA maps based on weighted and unweighted Unifrac distances showed that the use of HG did not reshape the structure of bacterial microbiota in the rumen fluid of cows. This result was not affected by the changes in rumen fermentation parameters mentioned above and the possible reasons for this need to be further elucidated in future research.

Previous studies have confirmed that *Prevotella* in the rumen typically ferments dietary hemicellulose, starch, and protein into succinate, propionate, and acetate. This also better explains why the increase in abundance of *Prevotella* species led to the production of more acetate and propionate salts in the rumen fluid of the HG group. The increase in abundance of *Prevotella* and *Bacteroidetes*, as typical Gram-negative and anaerobic bacteria, also indicates that the HG group has, to some extent, increased the abundance of Gram-negative and anaerobic bacteria. *Methanobrevibacter*, the main methane-producing microbe, positively correlates with methane production [43]. This experiment found that the HG group significantly reduced the abundance of *Methanobrevibacter*, suggesting that HG feeding may have a positive effect on methane emissions. However, this conclusion still requires a lot of experiments to prove. There is no clear report on the effect of *Lachnospiraceae_ND3007_group* on rumen fermentation, and further research is needed in the future. However, previous reports have shown that *Lachnospiraceae* provides energy to the body through carbohydrate metabolism in the rumen, with *Lachnospiraceae NK4A136 group* being propionic acid-producing bacteria [44,45]. *Lachnospiraceae_ND3007_group* may also have similar functions. The relative abundance of *Succiniblasticum* in this experiment is positively correlated with the yield of propionate and TVFA. This may be due to *Succiniblasticum,* specifically fermenting succinate and converting it into propionate [46]. In this experiment, LEfSe was used to analyze the relative abundance of microbial communities in the LG and HG groups, with LDA scores > 2. The results showed that *Stenotrophomonas* and *Xanthomonadaceae* were significantly enriched bacteria in the rumen fluid of cows in the HG group. According to relevant studies, the increase in abundance of *Stenotrophomonas* in the rumen and the translocation of LPS produced by Gram-negative bacteria from the rumen to the mammary gland are important endogenous factors that induce mastitis [34]. This also validates the results of Hu et al. [34] and explains why SARA may induce mastitis symptoms in cows. *Xanthomonadaceae* belongs to the *Proteobacteria*, which can utilize carbohydrates as a nutritional source for ruminants [47]. *Proteobacteria* can utilize the large amount of SCFAs produced by HG to provide energy for the body. Overall, HG feeding did not alter microbial diversity, but the composition of microbial communities at the phylum and genus levels changed and significantly increased the concentration of SCFAs in rumen fluid. This may be due to HG feeding promoting rumen fermentation by altering the microbial community structure.

Further analysis of microbial function using PICRUSt2 revealed that the core functional differences between the two treatment groups were concentrated in metabolic functions, mainly including the metabolism of co-factors and vitamins, carbohydrate metabolism, energy metabolism, and lipid metabolism. Firstly, compared to the other 15 metabolic pathways, cofactors and vitamin metabolism, carbohydrate metabolism, and amino acid metabolism have the highest microbial abundance. These pathways are crucial for the survival, growth, and reproduction of animal gastrointestinal microbiota [48]. Energy is an important component of feed, the most critical factor in animal feed intake, and an important substance for animals to survive, carry out life activities, and reproduce offspring [49]. Therefore, carbohydrate metabolism and energy metabolism are particularly important for animals. *Firmicutes*, *Bacteroidetes*, and *Prevotella* are associated with carbohydrate metabolism and energy metabolism [50]. *Ruminococcus* plays an important role in digesting resistant starch but is also associated with metabolic pathways in intestinal diseases, immune diseases, and neurological disorders [51]. This study indicates that the metabolism of cofactors and vitamins, carbohydrate metabolism, amino acid metabolism, energy metabolism, glycan biosynthesis and metabolism, and lipid metabolism in the HG group are significantly higher than those in the LG group. This indicates that with the increase in grain content in the diet, there is a promoting effect on the microbial metabolism in the rumen of cows.

## 5. Conclusions

This study indicates that a high-grain diet significantly reduces the milk fat content of lactating cows. In addition, the HG group promoted rumen fermentation by altering the microbial composition of lactating cows. The HG group had no significant effect on the alpha diversity of rumen bacterial communities in cows but could up-regulate the relative abundance of *Prevotella* and down-regulate the relative abundance of *Methanobrevibacter* and *Lachnospiraceae ND3007_group*. On the one hand, the HG group produces SCFAs as an energy source for the body by increasing the abundance of *Prevotella* and *Xanthomonadaceae*, and on the other hand, it increases the abundance of *Stenotrophomonas*, which has an adverse effect on the occurrence of mastitis in the body. These findings provide a theoretical basis for the relationship between the microbiota-host under HG feeding in lactating dairy cows.

## Figures and Tables

**Table 1:** Ingredient and chemical composition of the experimental diets.

Items	Low-Grain Diets (LG)	High-Grain Diets (HG)
Ingredient, % of DM		
Alfalfa hay	24	17
Oaten hay	24	17
Corn silage	12	6
Corn grain	16.6	31.5
Soybean meal	6.84	6.84
DDGS ^1^	3.7	4
Oatmeal	4	4
Rootlet	1.6	3.4
Spray corn husk	2	4
Corn germ meal	3	4
Premix ^2^	2.26	2.26
Total	100	100
Nutrient composition		
DM ^3^, %	50.74	51.03
Ash, % of DM	6.79	5.67
Crude protein, % of DM	15.43	15.54
Crude fat, % of DM	2.97	3.03
NDF ^4^, % of DM	38.02	31.08
ADF ^5^, % of DM	23.42	17.95
Ca, % of DM	0.84	0.85
P, % of DM	0.35	0.38
Starch, % of DM	17.36	29.72
NFC ^6^	36.79	44.68
NFC/NDF	0.97	1.44
NEL ^7^, Mcal/kg of DM	1.54	1.63

^1^ DDGS = Distillers Dried Grains with Solubles; ^2^ Each kg of premix contains 22,000 IU of vitamin A, 5500 IU of vitamin D3, 50,000 IU of vitamin E, 42 IU of vitamin K3; 65.0 mg of Zn, 4.5 mg of Mn, 120.5 mg of Fe, 75 mg of Cu, 0.55 mg of I, 0.35 mg of Co, and 0.45 mg of Se; ^3^ DM = Dry matter; ^4^ NDF = neutral detergent fiber; ^5^ ADF = Acid detergent fiber ^6^ NFC = 100 − (%CP + %Ash + %EE + %NDF); ^7^ Estimated based on the NRC (2001).

**Table 2:** Effects of HG on feed intake and lactation performance in dairy cows.

Item	Treatment ^1^		SEM ^2^	*p*-Value
	LG	HG		
DMI ^3^, kg/d	21.83	21.23	0.71	0.69
Milk yield, kg/d	16.79	17.85	0.75	0.50
ECM ^4^, kg/d	17.05	17.68	0.57	0.63
Milk composition				
Fat, %	4.02	3.75	0.03	0.04
Protein, %	3.48	3.59	0.06	0.36
Lactose, %	4.80	4.84	0.03	0.56
Total solids, %	16.51	16.25	0.20	0.53
SCC ^5^, ×10^3^/mL	221.10	245.83	17.91	0.52
MUN ^6^, mg/dL	15.73	16.61	0.26	0.09

^1^ Treatment: LG = Low-grain diet; HG = High-grain diet; ^2^ SEM = Standard error of means; ^3^ DMI = Dry matter intake; ^4^ ECM = Energy-corrected milk; ^5^ SCC = Somatic cell count; ^6^ MUN = Milk urea nitrogen.

**Table 3:** Effects of HG on ruminal fermentation characteristics in dairy cows.

Item	Treatment ^1^		SEM ^2^	*p*-Value
	LG	HG		
Rumen pH	6.03	5.56	0.09	<0.01
NH_3_-N (mg/dL)	12.34	13.12	0.88	0.46
TVFA ^3^ (mM)	108.91	143.08	4.08	<0.01
Acetate (mM)	65.18	81.14	1.92	<0.01
Propionate (mM)	24.28	38.47	2.45	<0.01
Butyrate (mM)	14.24	17.52	0.78	0.03
Isobutyrate (mM)	1.47	1.26	0.29	0.75
Valerate (mM)	2.07	2.66	0.20	0.16
Isovalerate (mM)	1.62	2.02	0.22	0.39
VFA profile (mol/100 mol)				
Acetate	59.88	56.71	0.78	0.03
Propionate	22.30	26.89	1.07	0.02
Butyrate	13.08	12.25	0.57	0.49
Isobutyrate	1.35	0.88	0.27	0.42
Valerate	1.90	1.86	0.16	0.91
Isovalerate	1.49	1.41	0.19	0.85
Acetate/Propionate	2.69	2.14	0.16	<0.01

^1^ Treatment: LG = Low-grain diet; HG = High-grain diet; ^2^ SEM = Standard error of means; ^3^ TVFA = Total volatile fatty acid.

## Data Availability

Sequences generated in the current study have been deposited in the NCBI Sequence Read Archive database (SRA; http://www.ncbi.nlm.nih.gov/Traces/sra/, accessed on 17 July 2024) under the accession number PRJNA1141126.

## Supplementary Materials

The following supporting information can be downloaded at https://www.mdpi.com/article/10.3390/ani14172522/s1: Figure S1: Number of common and unique OTUs of rumen bacteria in LG and HG groups; Figure S2: ROC curve analysis of OTUs number in rumen fluid samples; and Table S1: Raw data and clean data generated by 16s rRNA sequencing of rumen fluid.
