# Supplementary material for: Effects of High-Grain Diet on Performance, Ruminal Fermentation, and Rumen Microbial Flora of Lactating Holstein Dairy Cows
Source: Animals (Basel). 2024 Aug 30;14(17):2522. doi: 10.3390/ani14172522 (PMC11394336; doi:10.3390/ani14172522)
Supplement: Supplementary file 1 [file animals-14-02522-s001.zip › animals-3158675-supplementary.pdf]

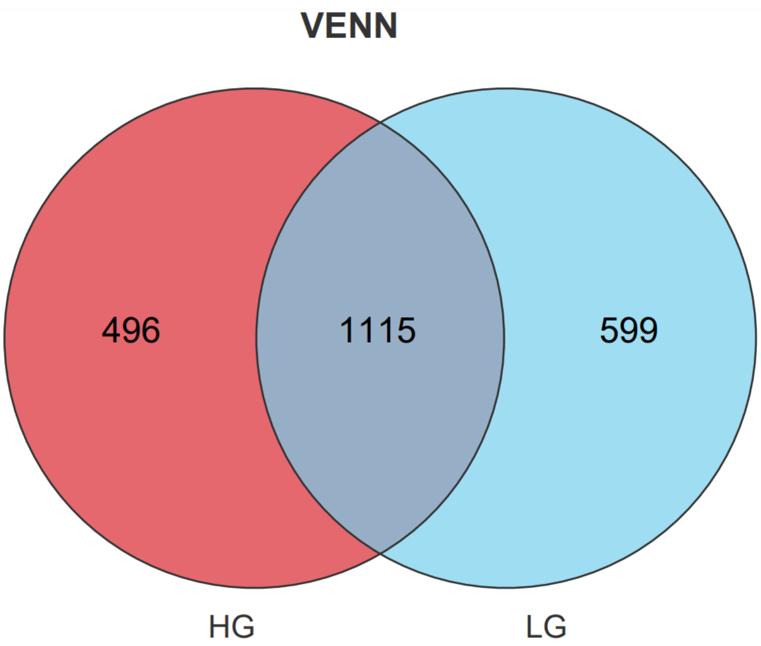

Supplementary Figure S1. Number of common and unique OTUs of rumen bacteria in LG and HG groups. LG = Low-grain diet; HG = high-grain diet.

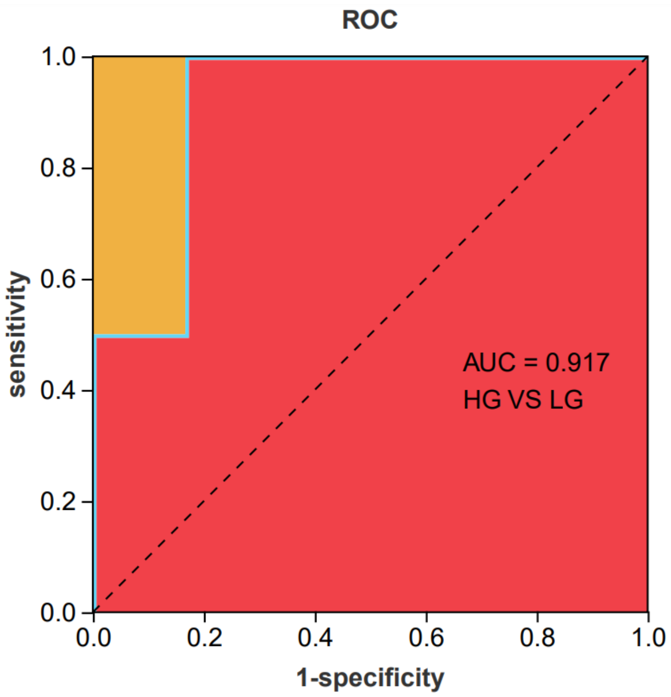

Supplementary Figure S2. ROC curve analysis of OTUs number in rumen fluid samples. LG = Low-grain diet; HG = high-grain diet

Supplementary Table S1.

Table S1 Raw data and clean data generated by 16s rRNA sequencing of rumen fluid

Sample Name	Raw Reads	Clean Reads	Raw Tags	Clean Tags	Chimera	Effective Tags	Effective Ratio (%)
LG-1	106422	106345	104736	104354	21096	83258	78.23
LG-2	110220	110155	108370	107845	22498	85347	77.43
LG-3	74626	74563	73330	73063	14026	59037	79.11
LG-4	113470	113376	111381	110916	22452	88464	77.96
LG-5	108679	108589	107005	106323	22242	84081	77.37
LG-6	110967	110880	109022	108582	22741	85841	77.36
HG-1	104965	104895	103145	102739	20153	82586	78.68
HG-2	111521	111453	109577	109174	22670	86504	77.57
HG-3	111172	111089	109147	108701	20643	88058	79.21
HG-4	73407	73361	72164	71878	14299	57579	78.44
HG-5	111379	111273	109255	108776	22148	86628	77.78
HG-6	107199	107122	105482	105045	22457	82588	77.04

LG = Low-grain diet; HG = high-grain diet
